# Erysipeloid cutaneous Leishmaniasis treated with the combination of metronidazole and clarithromycin

**DOI:** 10.1093/omcr/omae194

**Published:** 2025-03-20

**Authors:** Meryem Khallouki, Khaoula Jaatar, Layla Bendaoud, Mariem Aboudourib, Said Amal, Ouafa Hocar

**Affiliations:** Department of Dermatology, Faculty of Medicine and Pharmacy, Mohammed VI University Hospital 53 Ibn Sina Street, Cadi Ayad University, Marrakech 40000, Morocco; Department of Dermatology, Faculty of Medicine and Pharmacy, Mohammed VI University Hospital 53 Ibn Sina Street, Cadi Ayad University, Marrakech 40000, Morocco; Department of Dermatology, Faculty of Medicine and Pharmacy, Mohammed VI University Hospital 53 Ibn Sina Street, Cadi Ayad University, Marrakech 40000, Morocco; Department of Dermatology, Faculty of Medicine and Pharmacy, Mohammed VI University Hospital 53 Ibn Sina Street, Cadi Ayad University, Marrakech 40000, Morocco; Department of Dermatology, Faculty of Medicine and Pharmacy, Mohammed VI University Hospital 53 Ibn Sina Street, Cadi Ayad University, Marrakech 40000, Morocco; Department of Dermatology, Faculty of Medicine and Pharmacy, Mohammed VI University Hospital 53 Ibn Sina Street, Cadi Ayad University, Marrakech 40000, Morocco

**Keywords:** Leishmaniasis, phlebotomus, erysipeloid leishmanias

## Abstract

Leishmaniasis is caused by an intracellular parasite transmitted to humans by the bite of a sandfly: Phlebotomus. The disease can present in three ways: visceral, cutaneous, or mucocutaneous forms. Unusual clinical presentations of cutaneous leishmaniasis have been reported: psoriasiform, eczematiform, erysipeloid, and sporotrichoid, depending on host immune status and Leishmania subspecies. We report a case of an unusual presentation of erysipeloid cutaneous leishmaniasis treated with the combination of Metronidazole and Clarithromycin. A 67-year-old woman presented with a 2-month history of swelling of the centrofacial region, with an erythematous and edematous plaque; the episode was treated as facial erysipelas with antibiotics. In the absence of improvement, the diagnosis of cutaneous leishmaniasis in its erysipeloid form was suspected and then confirmed by a skin smear showing the presence of leishmania amastigotes. The patient was treated with metronidazole and clarithromycin for 30 days, with good progression.

## Introduction

Globally, cutaneous leishmaniasis (CL) affects 12 million cases and annually 2 million new cases occur. CL is endemic in Asia, Africa, parts of North and South America, and the Mediterranean region. Morocco is one of the endemic countries for infection [1]. Several forms of CL are described; they are mainly caused by Leishmania Major, *L. infantum*, and *L. Tropica*. The most common presentation is nodulo-ulcerative lesions localized on the exposed areas of the body. Other clinical presentations of CL have been reported such as psoriasiform, erysipeloid, and sporotrichoid forms, depending on the host immune status and the subspecies of Leishmania [2]. Several treatment options for CL are available. Pentavalent antimonials remain the first treatment option in most countries. The association metronidazole-clarithromycin is an effective therapeutic alternative in the treatment of CL. We report a case of an unusual presentation of erysipeloid cutaneous leishmaniasis treated with the combination of Metronidazole and Clarithromycin.

## Case report

**Figure 1:**
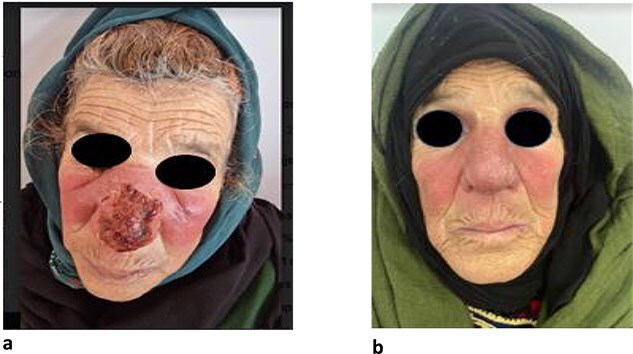
(a) Erythematous and edematous plaque covering the nose, cheeks, and reaching eyelids. (b) Regression of the lesions after treatment of the cutaneous erysipeloid leishmaniasis.

A 67-year-old woman from the region of Chichaoua which is an endemic area of Leishmania Tropica in Morocco. The patient presented with swelling in the centrofacial region that had been evolving for 2 months. She had a grandson treated for leishmaniasis. Clinical examination revealed erythematous and edematous plaque covering the centrofacial region and reaching the eyelids, associated with the presence of hemorrhagic crusts on the tip of the nose (Fig. 1a); the episode was treated as facial erysipelas with antibiotics (Amoxicillin-Clavulanic acid, ciprofloxacin) for 15 days without improvement. In front of anamnestic data and clinical examination, the diagnosis of CL in its erysipeloid form was strongly suspected and then confirmed by skin smear which had shown the presence of leishmania amastigotes. The identification of the *Leishmania* species is not available at our institution. The patient had left bundle branch block on the electrocardiogram, so we opted for the metronidazole-clarithromycin combination as an alternative to pentavalent antimonials. Our patient was treated with metronidazole 1,5 g daily and clarithromycin 15 mg/kg for 30 days with good progression (Fig. 1b).

## Discussion

The erysipeloid type is a rare and unusual presentation of CL, often leading to a late diagnosis [3]. Its frequency is estimated at 3%. This type usually affects elderly females and presented as erythematous infiltrative plaque over the face and resembling erysipelas [4]. The etiology of this type is unknown although factors such as senility, specific species of leishmania, hormonal changes, and changes in skin barrier with aging can cause such an unusual presentation [2]. Our patient is a female from an endemic region of leishmaniasis and is elderly, thus, she presented with all the epidemiological features. The diagnosis is confirmed by the presence of amastigotes on a Giemsa-stained smear. Pentavalent antimonials remain the first treatment option for CL in most countries. In addition, the metronidazole-clarithromycin combination has been described as an effective therapeutic alternative with better tolerability. Its efficiency approaches that of antimony salts (60 to 90%) and exceeds that of metronidazole alone [5]. As our patient had a contraindication to pentavalent antimonials; we opted for the metronidazole-clarithromycin combination. Metronidazole is used in the treatment of the infections caused by protozoa and most anaerobic bacteria. Following entry into anaerobic cells, metronidazole is metabolized. The toxic metabolites of metronidazole cause DNA damage [6]. Some studies on the treatment of experimental CL have demonstrated a significant reduction in lesion size and in the number of parasites via histological examination. Metronidazole is also a drug without major side effects, with easy administration, with low cost, and useful in the treatment of CL [7]. Claritromycin produces a direct effect on promastigotes and reduces the numer of host cells infected with parasites and the number of parasites in these cells. Combining metronidazole and clarithromycin is an attractive and tolerated therapeutic modality [8].
